# Role of the Triplet State and Protein Dynamics in the Formation and Stability of the Tryptophan Radical in an Apoazurin Mutant

**DOI:** 10.1021/acs.jpcb.2c02441

**Published:** 2022-08-17

**Authors:** Ignacio López-Peña, Christopher T. Lee, Joel J. Rivera, Judy E. Kim

**Affiliations:** Department of Chemistry and Biochemistry, University of California at San Diego, La Jolla, California92093, United States

## Abstract

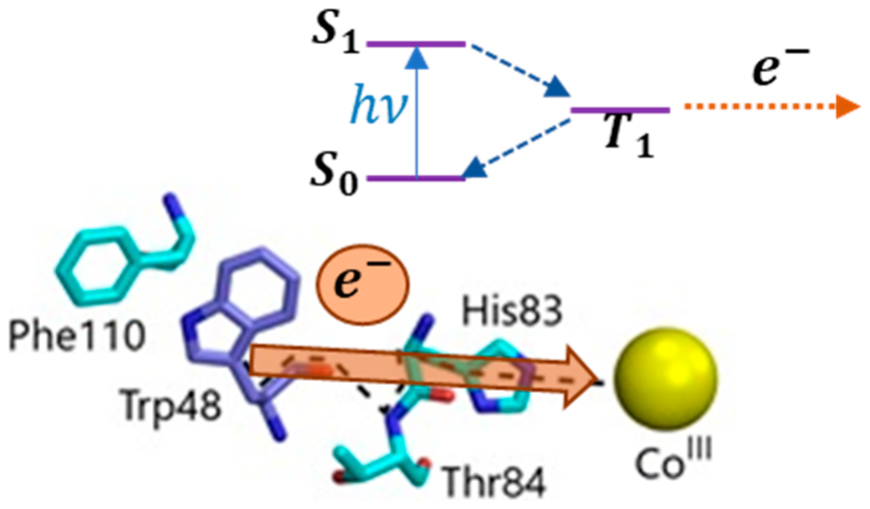

The protein, azurin, has enabled the study of the tryptophan radical. Upon UV excitation of tyrosine-deficient apoazurin and in the presence of a Co(III) electron acceptor, the neutral radical (W48•) is formed. The lifetime of W48• in apoazurin is 41 s, which is shorter than the lifetime of several hours in Zn-substituted azurin. Molecular dynamics simulations revealed enhanced fluctuations of apoazurin which likely destabilize W48•. The photophysics of W48 was investigated to probe the precursor state for ET. The phosphorescence intensity was eliminated in the presence of an electron acceptor while the fluorescence was unchanged; this quenching of the phosphorescence is attributed to ET. The kinetics associated with W48• were examined with a model that incorporates intersystem crossing, ET, deprotonation, and decay of the cation radical. The estimated rate constants for ET (6 × 10^6^ s^–1^) and deprotonation (3 × 10^5^ s^–1^) are in agreement with a photoinduced mechanism where W48• is derived from the triplet state. The triplet as the precursor state for ET was supported by photolysis of apoazurin with 280 nm in the absence and presence of triplet-absorbing 405 nm light. Absorption bands from the neutral radical were observed only in the presence of blue light.

## Introduction

Long-range electron transfer (ET) is an essential reaction in many biological processes, such as photosynthesis and aerobic respiration.^1^ In these reactions, the rate of ET is impacted by the kinetic barriers associated with long distances that are relevant to protein function. To overcome the barriers, proteins utilize multiple cofactors that act as redox-active intermediates; these intermediates enable rapid ET via a multistep mechanism (hopping).^2^ The redox-active cofactors can be metal centers, extrinsic organic cofactors, or the side chains of amino acids, for example, cysteine, tyrosine, and tryptophan.

The focus of the present work is the redox-active aromatic amino acid tryptophan. The oxidized radical intermediates of tyrosine and tryptophan play important roles in the reaction mechanisms of some enzymes.^3−6^ It has also been postulated that chains of tyrosine and tryptophan residues provide a charge transfer pathway that protects proteins from oxidative damage associated with enzymatic catalysis.^7,8^ Despite the importance of tyrosine and tryptophan radicals, investigation of these transient species remains challenging.

Several studies have characterized the tryptophan neutral radical in a blue-copper protein, azurin, of *Pseudomonas aeruginosa*.^9−15^ Azurin is a 128-residue protein with eight β-strands arranged in a Greek key motif and a small α-helical segment. The metal-binding site is located at one end of the protein, with a single native tryptophan residue (W48) buried in a hydrophobic pocket. Azurin is well-known for its remarkable thermodynamic stability^16−18^ and for the striking blue-shifted emission of W48.^19^

Studies on W48 revealed that the tryptophan radical can be photogenerated via a covalently bound photoactive label^9^ or direct UV-excitation of the Zn(II)-substituted protein (Zn-azurin).^12−14^ The mechanism for formation is expected to involve photoejection of an electron from closed-shell tryptophan to form the cation radical, followed by proton transfer to generate the neutral radical. The resulting neutral radical in a tyrosine-deficient Zn-azurin is a long-lived species that is stable for several hours in an oxygen-free environment.^13^ This long-lived species is only present in Zn-azurin and not the native holoprotein of Cu(II)-azurin.^13^ The long-lived nature of the neutral radical has led to its characterization by absorption, resonance Raman, and EPR spectroscopy.^12^ The radical is also generated in wild-type Zn-azurin that contains the two native tyrosine residues; however, the yield for formation is lower in wild-type azurin compared to the tyrosine-deficient mutants on account of tyrosine-to-tryptophan ET.^13^

The efficient generation of a stable neutral radical of W48 in azurin is remarkable, and with the exception of a tryptophan radical that persists for minutes in ribonucleotide reductase,^5^ the type of long-lived neutral radical observed in azurin has not been reported in other systems. The unique photophysics and environment of W48 may provide clues to the mechanism of radical formation in azurin. The fluorescence and phosphorescence properties of W48 have been studied extensively in native holoazurin, metal-substituted azurins, and apoazurin. The fluorescence intensity and lifetime are decreased in holoazurin compared to apoazurin.^19−25^ Metal-substituted azurins exhibit different degrees of fluorescence quenching that depends on the identity of the metal. It is known that the fluorescence quenching mechanism of W48 involves a fast intramolecular excited-state ET to the metal. In holoazurin, the theoretical rate constant for intramolecular ET between the tryptophan singlet excited state and Cu(II) center (10^9^ s^–1^) is larger than the rate constant for fluorescence (10^8^ s^–1^).^23^ The properties of the triplet state of W48, including the long triplet lifetime of milliseconds,^26,27^ have also been investigated. Variation in phosphorescence intensity of different metal-substituted azurins may reflect differences in the inherent lifetimes; alternatively, the quenching dynamics may also reflect intramolecular ET from the triplet excited state to the metal.^28^ For *inter*-molecular ET, when the rate constant for ET is smaller than the rate constant for fluorescence, one expects the triplet state to be the most probable precursor of photogenerated tryptophan radical. Because W48 in apoprotein exhibits relatively long excited singlet (∼ns) and triplet (∼ms) state lifetimes compared to the holoprotein, apoazurin is an excellent variant for studies of the mechanisms of intermolecular ET of azurin.

An important goal of the present study is to elucidate the precursor state for ET of W48 in apoazurin. The photophysics of apoazurin is evaluated in the presence and absence of an electron acceptor, and the kinetics of formation of the neutral radical is examined with a model that includes the rates of excitation, intersystem crossing, ET, deprotonation, and decay of cation radical. Molecular dynamics simulations complement the experimental results and shed light on the origin of the different stabilities of the radical in apo- and Zn-azurin. The results illustrate the role of the long-lived triplet state in intermolecular ET as well as the impact of protein dynamics on the stability of the neutral radical.

## Materials and Methods

### Sample Preparation

The recombinant azurin mutant Y72F/Y108F from *Pseudomonas aeruginosa* was expressed and purified as described previously^9,10^ with modifications.^13^ The single-tryptophan apoprotein mutant was generated from the holoprotein using a cyanide procedure^29^ and stored in 50 mM acetate buffer at pH 4.5. The apoprotein is referred to as apoAzW48. The ratio of absorbance at 630 to 280 nm of the purified apoprotein was less than 0.003. All experiments were performed in 20 mM phosphate buffer at pH 7.3. An aliquot of stock 10.0 mM aqueous solution of the exogenous electron acceptor [Co(NH_3_)_5_Cl]^2+^ was added to azurin samples when appropriate. *N*-Acetyl-l-tryptophanamide (NATA) was prepared as a 0.1 mM aqueous, buffered (phosphate, pH 7.2) stock solution for fluorescence quantum yield measurements. The reagents were obtained from the following commercial sources and used without purification: K_2_HPO_4_ and KH_2_PO_4_ salts from Fisher Chemical; [Co(NH_3_)_5_Cl]Cl_2_ (98%) from Sigma-Aldrich; KCN (96%) from Spectrum Chemical; NaCH_3_COO (99%) and CH_3_COOH (99%) from Fisher Chemical; CuSO_4_ (99%) from Alfa Aesar; and NATA (98%) from Sigma-Aldrich.

### Fluorescence and Phosphorescence Measurements and Quantum Yields

Emission of deoxygenated apoAzW48 samples was collected at room temperature on a Fluorolog 3–11 fluorometer (JY-Horiba) using a 10 mm × 2 mm fused silica cuvette. The samples were excited with 270 nm light along the 10 mm path length, and emission from 275 to 535 nm was collected in a right-angle geometry along the 2 mm path length. The emission and excitation monochromator slits were set to a bandpass of 2 nm. The emission spectra were integrated for 0.8 s per step of 2 nm.

The fluorescence and phosphorescence quantum yields were determined using 10–15 μM NATA as the standard with known fluorescence quantum yield, Φ_fluo_, of 0.13 in pH 7.2.^30^ The azurin spectra were normalized for light absorbed by the solution and, if relevant, the fractional absorption from W48 relative to all absorbing species. The spectra were additionally normalized for the integrated emission from NATA (300–500 nm) acquired on the same day. These normalized curves were integrated to determine Φ_fluo_ (275–400 nm), and Φ_phos_ (400–535 nm) for azurin. The sample of NATA was not deoxygenated; the quantum yields in the absence and presence of O_2_ were within the error of the measurement.

### Photolysis and Kinetics Measurements

Samples of apoAzW48 in phosphate buffer, pH 7.3, were deoxygenated on a Schlenk line using multiple cycles of pump-purge with argon gas. The deoxygenated solutions were continuously irradiated with 280 nm (0.97 mW), 405 nm (440 mW), or both wavelengths in the presence and absence of the electron acceptor [Co(NH_3_)_5_Cl]^2+^; when included, there was an excess of [Co(NH_3_)_5_Cl]^2+^ by a factor of 2. This 2-fold excess of the electron acceptor ensured maximum yield of formation of the neutral tryptophan radical based on concentration-dependence studies in our lab.

The UV beam from a light emitting diode LED280J OPTAN (Crystal IS, New York) produced light centered at 280 nm with a full-width at half-maximum (fwhm) range of 12 nm as reported by the manufacturer. The average light output was 0.97 mW. The ball lens integrated into the LED housing shaped and directed the UV beam perpendicular to the probe beam of a scanning spectrophotometer (Shimadzu UV-3600). The UV beam produced an 8 × 8 mm squircle image, which was overlapped with the probe beam (10–16 mm in height) at their intersection in the 10 mm × 2 mm fused silica cuvette. The 405 nm beam was provided by a Blu-Ray laser diode, harvested from a Pioneer BDR-209DBK drive. This light was focused with an AR-coated lens and made roughly collinear to the UV beam. The average output power of this diode was 450 mW. The beam size was similar to the UV beam at the sample.

Samples were continuously illuminated with actinic light of 280 nm, 405 nm, or both wavelengths along the 2 mm sample path length of the square 2 × 10 mm fused silica cuvette. Absorption spectra were recorded while 280 nm and/or 405 nm light was incident on the sample. Typical incident powers at the sample were 0.80, 0.63, 0.54, 0.38, 0.30, or 0.10 mW (280 nm) and 440 mW (405 nm). During experiments with 405 nm, a 405 nm long pass filter (Semrock, New York) was placed after the sample along the probe light path of the spectrophotometer. A long-pass filter was not necessary for 280 nm-only photolysis experiments when spectra were acquired above 285 nm. The absorbance was measured through the 10 mm path of the cell using a 2 nm spectral bandpass. A schematic of the photolysis setup is provided in Supporting Information. Kinetic traces were acquired in single-wavelength mode, where the absorbance at 514 nm was collected at 0.1 s intervals in the absence of actinic light, and with continuous excitation with 280 or 405 nm or both wavelengths simultaneously. In these kinetic traces, a slight drift in the baseline absorbance at 514 nm was recorded; this drift was small and linear, and the slope of the drift (<0.001 absorbance in 300 s) was used to correct the kinetic traces. Samples were not stirred during photolysis.

### Determination of Yields and Kinetic Rate Constants

#### Yield of Formation of W48•

The quantum yield for formation of radical, Φ_rad_, was calculated^13^ from the rate of formation of radical, rate_form_(W48•), and rate of formation of singlet excited state, rate_form_ (W48*):
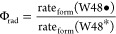
1

The rate of formation of radical was determined from the initial change in absorbance of the sample at 514 nm (using ε_514 nm_ = 2200 M^–1^ cm^–1^)^13^ in the illuminated volume. The rate of formation of excited singlet states was calculated from the absorbed light assuming each photon absorbed by W48 results in formation of W48*. The absorbed light in photons per second took into account the presence of the absorbing species, namely, [Co(NH_3_)_5_Cl]^2+^ (ε_280_ = 550 M^–1^ cm^–1^) and phenylalanine residues in the protein (ε_280_ = 250 M^–1^ cm^–1^). Thus, the expression for absorbed light by W48 is the product of total absorbed light by the entire solution and fractional contribution from W48 relative to all absorbing species.^13^ Evolution of the absorbance at 280 nm during the experiment could impact the quantum yield for radical formation. The change in absorbance of the sample because of depletion of tryptophan and [Co(NH_3_)_5_Cl]^2+^ and growth of neutral radical at 280 nm was less than 2.5% of the absorbance value of ∼0.12 during the experiment, and thus, no corrections were made to reflect changes in the absorbance at 280 nm during the course of an experiment.

#### Rate Constants for W48•

The rate constants for ET, deprotonation, and radical decay were obtained from kinetic traces associated with W48•. The fractional population of W48• was expressed via eq 2, where [W48]_0_ is the initial concentration of W48 in the sample, as measured at 292 nm:
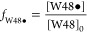
2

To gain insight into the photooxidation mechanism, kinetic traces of *f*_W__48•_ were fit to a set of ordinary differential equations for the photooxidation reaction described by the following scheme:

W48 represents the starting, ground-state tryptophan residue at position 48 of apoAzW48 that is photoexcited with rate constant *k*_excit_ to generate W48*, the singlet excited state, which can undergo intersystem crossing to form ^3^W48*, the triplet excited state, with quantum yield for triplet formation, Φ_isc_, and triplet lifetime in the absence of acceptor, τ_*T*_.

The excitation rate constant is given as
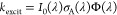
3In this equation, *I*_0_(λ) is the incident photon flux in units of photons s^–1^ cm^–2^. Note that an inner filter (IF) term, 10^–Abs_IF_^, where Abs_IF_ is the absorbance of the other absorbing species at 280 nm for the excitation path length, was not applied because IF correction was less than 3% for all powers. The other terms in eq 3 are σ_A_(λ), the absorption cross-section of W48 in units of cm^2^ molecule^–1^, and Φ(λ), the probability for generation of W48* per incident photon (assumed to be 1). For the highest UV power of 0.80 mW, the photon flux *I*_0_(λ) was 1.8 × 10^15^ photons s^–1^ cm^–2^. The value for σ_A_(λ) is derived from σ_A_(λ) = ε(λ) × (3.824 × 10^–21^ mol L^–1^ cm^3^), where ε(λ) reflects only W48 and not the other absorbing species of phenylalanine (ε(280) for ZnAzW48 is 6690 M^–1^ cm^–1^ and ε(280) for the all-phe variant with the additional mutation of W48F is 250 M^–1^ cm^–1^).^13^ Thus, assuming apoAzW48 and ZnAzW48 have the same values of molar absorptivity, ε(280) for W48 is 6440 M^–1^ cm^–1^ and σ_A_ (280) for W48 in AzW48 is 2.46 × 10^–17^ cm^2^ molecule^–1^. There are six experimental values for *k*_excit_ based on the incident power in the known illuminated area.

Upon photoexcitation, W48* undergoes intersystem crossing (*k*_isc_) or decays to the ground state via radiative (*k*_rad_) or other nonradiative (internal conversion, *k*_ic_) paths. Collectively, these three paths give rise to the observed rate constant for fluorescence, *k*_fluo_ = *k*_rad_ + *k*_ic_ + *k*_isc_. The quantum yield for intersystem crossing, Φ_isc_, is given by Φ_isc_ = *k*_isc_/*k*_fluo_, and thus the value of *k*_isc_ can be determined from *k*_isc_ = Φ_isc_*k*_fluo_. The value of *k*_fluo_ is 3.3 × 10^8^ s^–1^ based on τ_fluo_ = 3.0 ns, which is the weighted lifetime for the wild-type apoprotein.^31,32^ A value of Φ_isc_ = 0.3 was used, described below, resulting in *k*_isc_ = 9.9 × 10^7^ s^–1^. With knowledge of *k*_isc_, the sum of rate constants for the remaining pathways can be determined: *k*_rad_ + *k*_ic_ = *k*_fluo_ – *k*_isc_ = 2.3 × 10^8^ s^–1^.

In Scheme I, there are separate pathways for loss of W48•^+^ (rate constant *k*_decay_^′^) and loss of W48• (rate constant *k*_decay_) that result in the formation of unproductive photoproducts, collectively referred to as W48^X^, that are not detectable at 514 nm. In this scheme, W48^X^ encompasses all possible photoproducts that may result from W48^•+^ and W48•. There is an additional pathway for depletion of W48•^+^ via back-ET from other reducing species (e.g., sulfur-containing amino acids) to form the starting W48 (rate constant *k*_back_). The set of differential eqs (Supporting Information) was solved numerically for [W48^•^] and fit to a set of kinetic traces that monitored *f*_W__48•_ as a function of time for various excitation (280 nm) powers. The initial condition was *f*_W__48•_ = 0 because 100% of molecules are in the closed-shell, ground state at *t* = 0.

**Scheme I:**
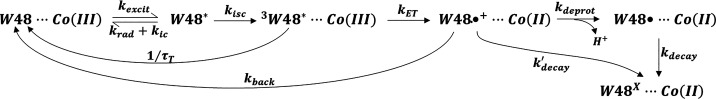
Photooxidation of W48 in AzW48 with Co(III) as Electron Acceptor The interacting electron donor and acceptor pair is denoted W48···Co(III). The tryptophan singlet excited state, triplet excited state, cation radical, and neutral radical are denoted W48*, ^3^W48*, W48•^+^, and W48•, respectively. The unreactive oxidized tryptophan photoproducts are denoted W48^X^. The value of *k*_excit_ is determined experimentally; fixed rate constants are *k*_isc_ = 9.9 × 10^7^ s^–1^, *k*_rad_ + *k*_ic_ = 2.3 × 10^8^ s^–1^, 1/*τ*_*T*_ = 1.9 s^–1^, and *k*_decay_ = 0.024 s^–1^. See main text for details.

The value of *k*_excit_ was determined from the incident power in the known illuminated area (eq 3); τ_fluo_ = 3.0 ns was based on literature;^31,32^ and τ_*T*_ = 0.53 s and *k*_decay_ = 0.024 s^–1^ were fixed and determined by the experiment (see below). The value of Φ_isc_ = 0.3 was based on the experimentally determined maximum quantum yield for formation of W48• in ZnAzW48. This value is consistent with the literature values for Φ_isc_ for tryptophan compounds. Reported values include 0.10 for tryptophan,^33^ 0.15–0.23 for NATA,^34,35^ and up to 0.43 (cyclohexane)^36^ and 0.3 (supersonic free jet) for indole.^37^ The use of Φ_isc_ = 0.2 gave similar results (see below). The rate constants *k*_ET_, *k*_deprot_, *k*_back_, and *k*_decay_^′^ were determined through global least-squares fitting of the data to the set of differential equations using IGOR Pro 8.

### Molecular Dynamics (MD) Simulations

#### System Preparation

The structure of apoAzW48 was modeled using crystallographic coordinates of wild-type apoazurin (PDB ID: 1E65)^38^ obtained from the Protein Data Bank. While the mutant in the current study differs from wild-type in terms of the two native, solvent-exposed tyrosine residues, it is expected that the MD results will be similar for wild-type and the mutant, especially in the regions of interest. The coordinates were processed to predict the protonation state of titratable residues at pH 7 using the H++ web server.^39−41^ All His residues were protonated in the neutral state, and residues that form metal ligands, H46 and H117, were protonated at Nε. The X-ray resolved disulfide bridge between residues 3 and 26 was intact. The protein was modeled using the AMBER 14SB force field.^42^ The structure was solvated with 15100 TIP3P water molecules in a box with initial dimensions 84 × 74 × 82 Å and neutralized by addition of three Na^+^ ions. The structure of wild-type Zn-azurin (PDB ID: 1E67)^43^ was prepared similarly. The Zn^2+^ ion and its ligands, G45, H46, C112, and H117, were parametrized using Metal Center Parameter Builder (MCPB), as described by Li and Merz.^44^

#### Simulation

All MD simulations were performed using AMBER software version 18.^45^ For each system, we performed restrained minimization followed by gradual heating to the target temperature of 300 K using a Berendsen thermostat (NVT). A Berendsen barostat was used to bring each system to the target pressure of 1 atm (NPT). Subsequently, each system was equilibrated for 0.5 ns under constant temperature and pressure conditions (NPT) with protein backbone restraints. Following this equilibration, the backbone restraints were released, and an additional 1 ns of equilibration at NPT was run. For all systems, hydrogen-mass repartitioning was employed to achieve 4 fs time steps with rigid hydrogen bonds enforced by the SHAKE algorithm.^46^ A nonbonded cutoff of 10 Å was used for all steps with the Particle Mesh Ewald method. In minimization, a harmonic restraint of 10.0 kcal/mol Å^2^ on backbone atoms was applied. A harmonic restraint of 5.0 kcal/mol Å^2^ was used in the restrained heating and equilibration steps. For the unrestrained equilibration and production steps, the temperature was controlled using a Langevin thermostat with a collision frequency of 5 ps^–1^. Constant pressure was enforced in unrestrained equilibration and production steps using the Monte Carlo barostat with a reference pressure of 1 atm, relaxation time of 2 ps, and 1000 steps between checks. The trajectories were extended in production runs to 3 μs for each system totaling 6 μs across all systems. The postequilibration trajectories were analyzed using VMD,^47^ PyMOL,^48^ PYTRAJ,^49,50^ and MDTraj.^51^ The structural flexibility was assessed by calculating the root-mean-square fluctuation (RMSF) of Cα atoms after superimposing the trajectory onto the first frame.

## Results

### Emission of apoAzW48 with and without [Co(NH_3_)_5_Cl]^2+^

**Figure 1:**
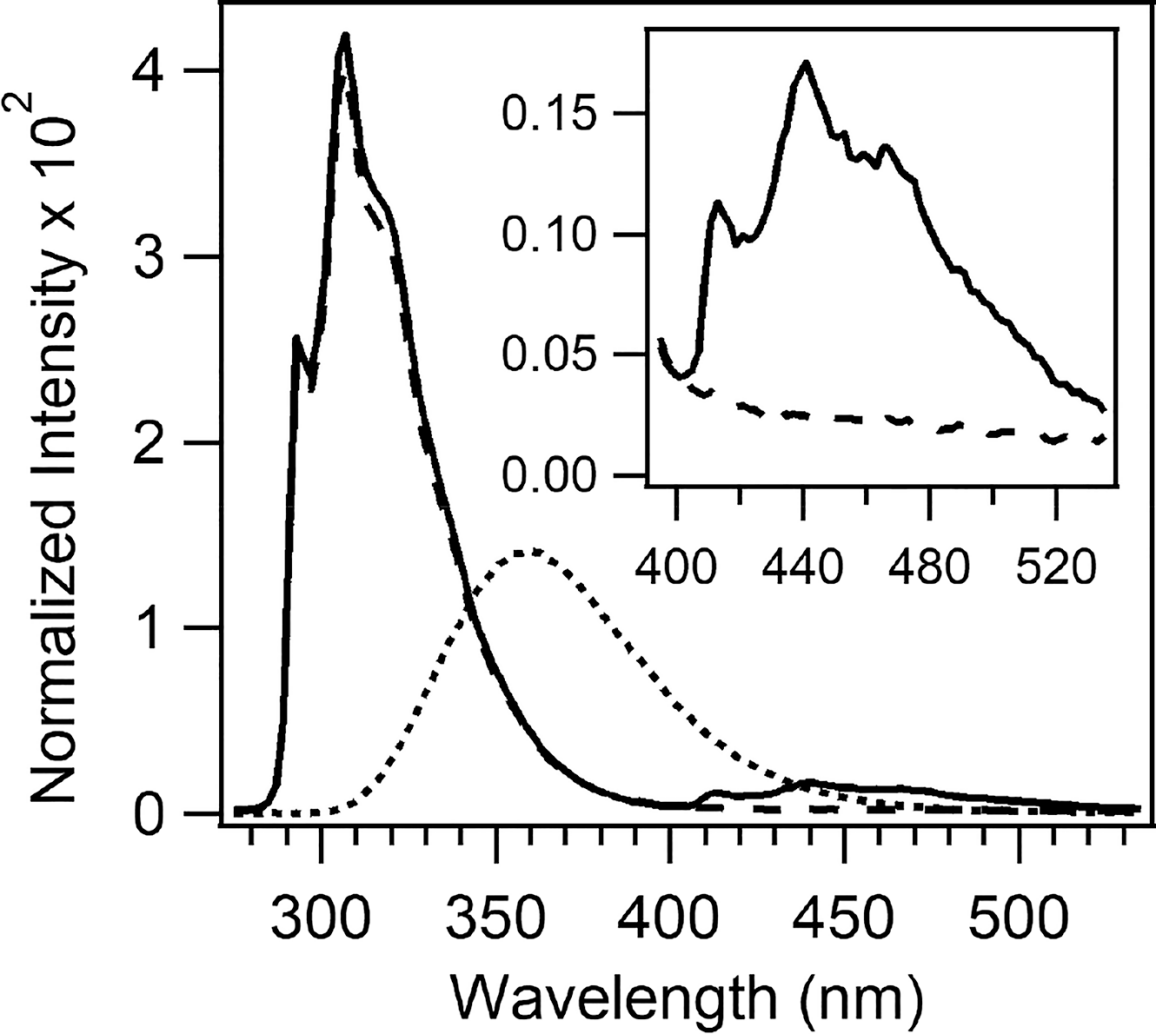
Normalized fluorescence spectra of solutions of deoxygenated 11 μM apoAzW48 in the absence (solid) and presence (dashed) of 22 μM [Co(NH_3_)_5_Cl]^2+^. The emission from NATA (dotted) is also shown as reference. Inset shows an enlarged view of the protein phosphorescence region 400–535 nm.

The quenched phosphorescence in protein samples may reflect intermolecular ET from the triplet excited state of tryptophan to exogenous electron acceptors.^52−54^ To investigate the possibility that the triplet state is the parent state for the tryptophan radical, we examined the room-temperature phosphorescence of deoxygenated apoAzW48 in the presence of the irreversible Co(III) electron acceptor, [Co(NH_3_)_5_Cl]^2+^. Figure 1 compares the emission spectra of apoAzW48 in the absence and presence of Co(III). The value of apoAzW48 Φ_fluo_ was 0.21 ± 0.01 and Φ_phos_ was 0.015 ± 0.001 in the absence of Co(III). In the presence of Co(III), Φ_fluo_ was 0.20 ± 0.01 and Φ_phos_ was not measurable (<0.001). The shape of the fluorescence spectra of apoAzW48 was unaffected by the presence of Co(III), but the phosphorescence emission above 400 nm vanished in the presence of Co(III); the presence of Co(III) did not affect the shape or intensity of NATA emission (Supporting Information).

### Triplet Absorption of apoAzW48

**Figure 2:**
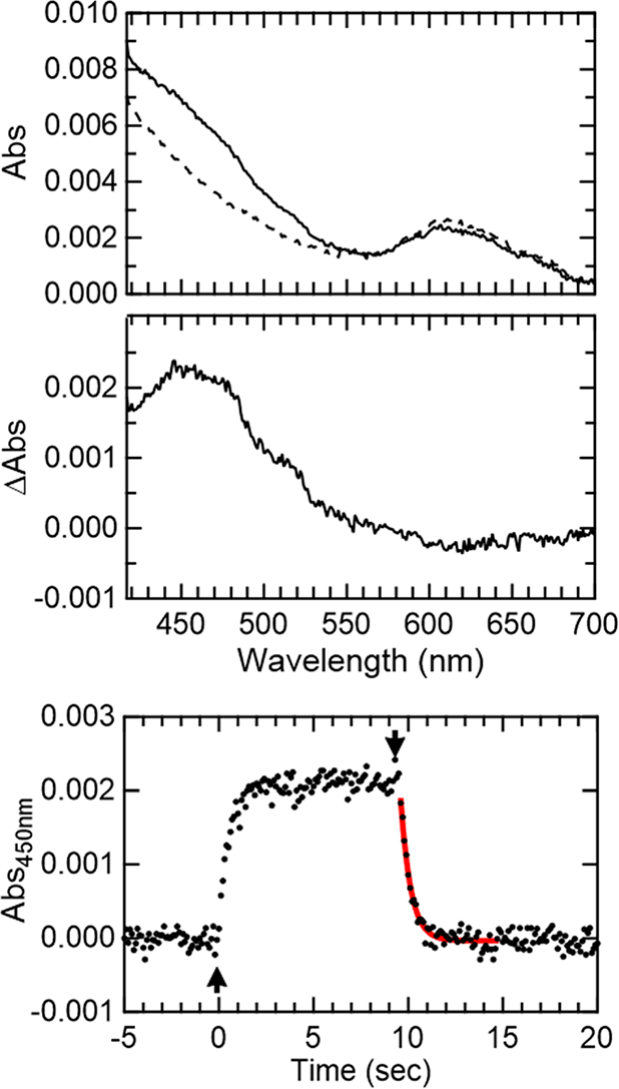
Absorption of a solution of deoxygenated 250 μM apoAzW48 during 280 nm photolysis. The electron acceptor [Co(NH_3_)_5_Cl]^2+^ was absent from the sample. Top: Absorption spectrum before photolysis (dashed line), and during 280 nm photolysis with incident power 0.97 mW (solid line). Middle: The difference spectrum after subtraction of the prephotolysis spectrum from the 280 nm continuous-photolysis spectrum. Bottom: Kinetics of the absorbance at 450 nm with incident 280 nm power 1.1 mW. The arrows mark the time at which the 280 nm light was turned on (↑) and off (↓). The absorbance prior to turning on the 280 nm light was 0.047, and this offset value was subtracted from the data. The red curve is a fit to the decay using a monoexponential function. The absorption spectra and kinetic trace were acquired with two different samples.

To investigate the triplet state of apoAzW48, the absorption spectrum was measured during continuous excitation with 280 nm. Figure 2 (top panel) shows that apoAzW48 developed a light-induced change in absorbance in the absence of Co(III) and O_2_. The middle panel displays the difference spectrum in which the prephotolysis spectrum was subtracted from the spectrum of the sample that was continuously excited with 280 nm. The difference spectrum displays an increase in the absorbance at 450 nm, and a decrease at 630 nm. The absorption band at 450 nm is similar to the transient absorption assigned to the triplet–triplet transition of l-trp^55^ as well as the transient absorption assigned to tryptophan triplet in parvalbumin.^56^ The decay of the 450 nm absorbance was monitored upon turning off the 280 nm light, and the resulting decay curve was fit to a single exponential curve; the fit is shown in the bottom panel of Figure 2. The time-constant for decay, τ_*T*_, is 530 ± 20 ms (*n* = 5 trials), which is consistent with tryptophan phosphorescence decay lifetime measurements of 444^27^ and 600 ms^26^ in wild-type apoazurin. The slight absorbance bleach at 630 nm is a known feature of Cu(II)AzW48 caused by the reduction of trace amounts of Cu(II)-azurin to Cu(I)-azurin in the nominal apoAzW48 sample. The fraction of Cu(II)AzW48 contaminant is less than 0.15% based on ε_628_ = 5900 cm^–1^ M^–1^. The small shoulder at 515 nm is a feature of the tryptophan neutral radical, which likely reflects the presence of trace amounts of both ZnAzW48 and Cu(II)AzW48; this combination has been shown to generate neutral radical.^13^

### Photolysis of Triplet apoAzW48

**Figure 3:**
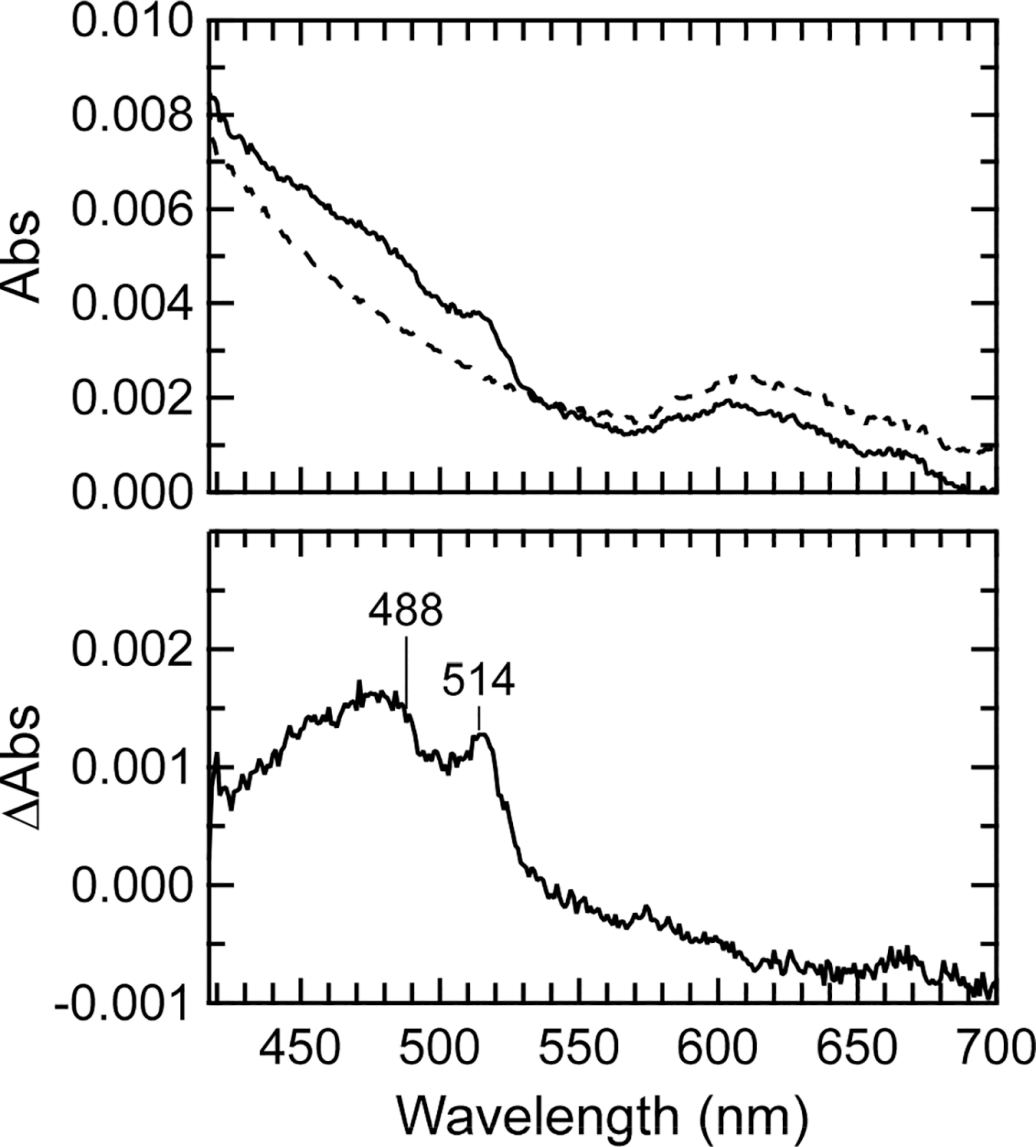
Absorption spectra of a solution of deoxygenated 250 μM apoAzW48 during simultaneous 280 + 405 nm photolysis. The electron acceptor [Co(NH_3_)_5_Cl]^2+^ was absent from the sample. Top: absorption spectrum before photolysis (dashed line), and during simultaneous 280 and 405 nm photolysis (solid line). Bottom: the difference spectrum after subtraction of the prephotolysis spectrum from the continuous-photolysis spectrum. The indicated new peaks are assigned to the neutral radical.

The photooxidation of triplet excited states can occur via direct light-induced ionization.^57^ To test the possibility that photoionization of the tryptophan triplet generates neutral radical, deoxygenated apoAzW48 samples in the absence of Co(III) were simultaneously exposed to 280 and 405 nm light; the 405 nm excitation overlaps the absorption band of the tryptophan triplet. The top panel of Figure 3 shows the absorption spectrum of the sample before and during simultaneous photolysis with 280 and 405 nm. The difference spectrum shown in the bottom panel of Figure 3 indicates an increase in absorbance at 488 and 515 nm during photolysis, and these peaks correspond to the tryptophan neutral radical. The peaks disappeared when the 280 and 405 nm sources were turned off. These features that appeared with simultaneous irradiation with 280 and 405 nm are different from those observed with only 280 nm radiation (Figure 2). Upon irradiation of apoAzW48 with only 405 nm, no new spectral features were generated, and instead, the absorbance baseline of the instrument was offset (Supporting Information).

### Generation of apoAzW48•

**Figure 4:**
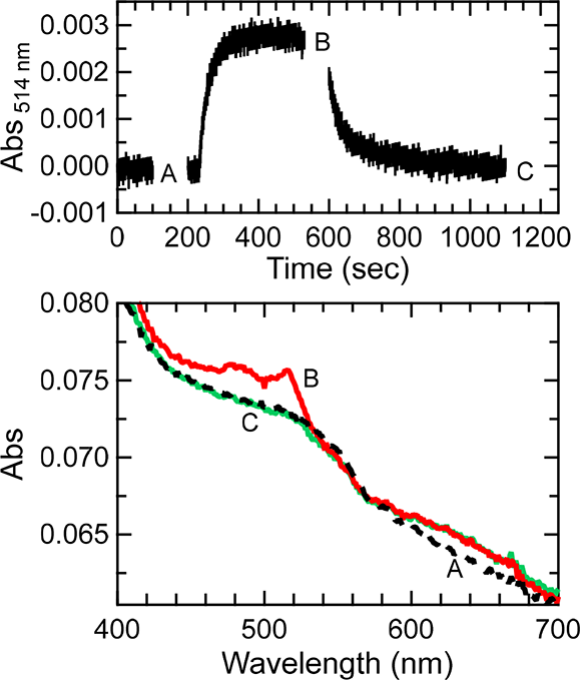
Photolysis of a solution of deoxygenated 75 μM apoAzW48 in the presence of 150 μM [Co(NH_3_)_5_Cl]^2+^. Top panel shows absorption at 514 nm before exposure with 280 nm (time 0 to 230 s), while 280 nm light was incident on the sample (time 230 to 600 s), and after the 280 nm light was turned off (time 600 to 1100 s). Absorption spectra acquired at time points indicated A, B, and C are shown in the bottom panel before (spectrum A in dashed black), during (spectrum B in solid red) and after (spectrum C in solid green) 280 nm photolysis. Spectra B and C were corrected for minor baseline drift (less than 0.001 absorbance per 300 s).

**Figure 5:**
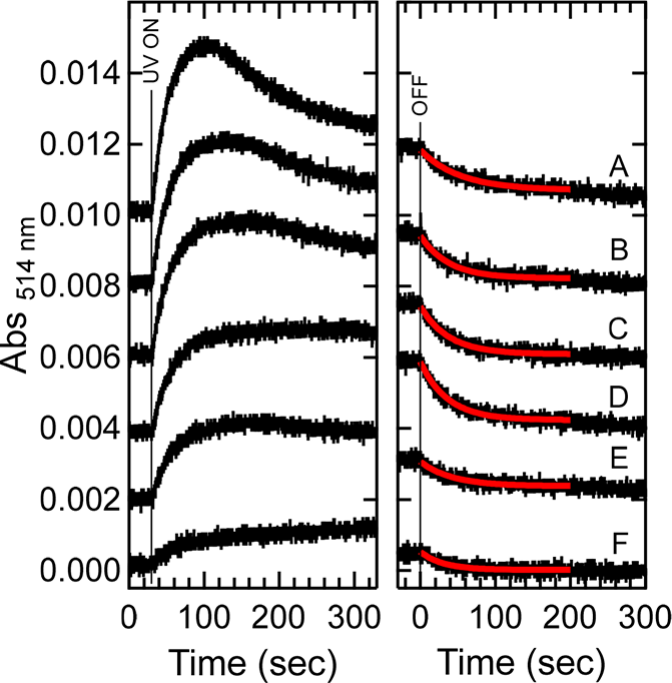
Photolysis of a solution of deoxygenated 75 μM apoAzW48 in the presence of 150 μM [Co(NH_3_)_5_Cl]^2+^ with various incident flux. The left panel shows the absorption at 514 nm upon turning on the 280 nm UV light. After 330 s, the instrument was switched to absorption mode and spectra were collected. The instrument was then reprogrammed to kinetics mode, and the right panel shows the decay after the light source is turned off. The decays were fit to monoexponential functions, shown in red. Labels A–F correspond to incident photon flux: 1.8 × 10^15^, 1.4 × 10^15^, 1.2 × 10^15^, 8.4 × 10^14^, 6.6 × 10^14^, and 2.2 × 10^14^ photons s^–1^ cm^–2^, respectively; the corresponding incident powers were 0.80, 0.63, 0.54, 0.38, 0.30, and 0.10 mW, respectively. The traces in both panels were offset (A) 0.010, (B) 0.008, (C) 0.006, (D) 0.004, (E) and 0.002 absorbance units for clarity.

Photolysis experiments of deoxygenated apoAzW48 in the presence of the Co(III) electron acceptor were pursued. Figure 4 shows the change in absorbance at 514 nm during continuous 280 nm excitation; absorption spectra before, during, and after 280 nm excitation are also shown. The absorbance at 514 nm increased when the 280 nm source was turned on, and decayed when the UV light was turned off. The absorption spectrum during photolysis displays features of the neutral radical at 488 and 515 nm. The kinetics of the formation of the radical as a function of light flux was also investigated. In Figure 5, the left panel shows the dependence of the absorption at 514 nm on the incident UV (280 nm) light flux. The appearance of an inflection suggests that a product that does not absorb 514 nm light is formed (e.g., decay of the cation and/or neutral radical, see below). In the right panel, the decay curves upon turning off the UV source are shown, including fits to monoexponential functions, which revealed a rate constant *k*_decay_ = 0.024 ± 0.003 s^–1^ (*n* = 6) for the neutral radical. This decay corresponds to a lifetime of the neutral radical in apoAzW48 of 41 ± 6 s, which is significantly shorter than the lifetime of the neutral radical reported for ZnAzW48 (∼7 h).^13^

**Figure 6:**
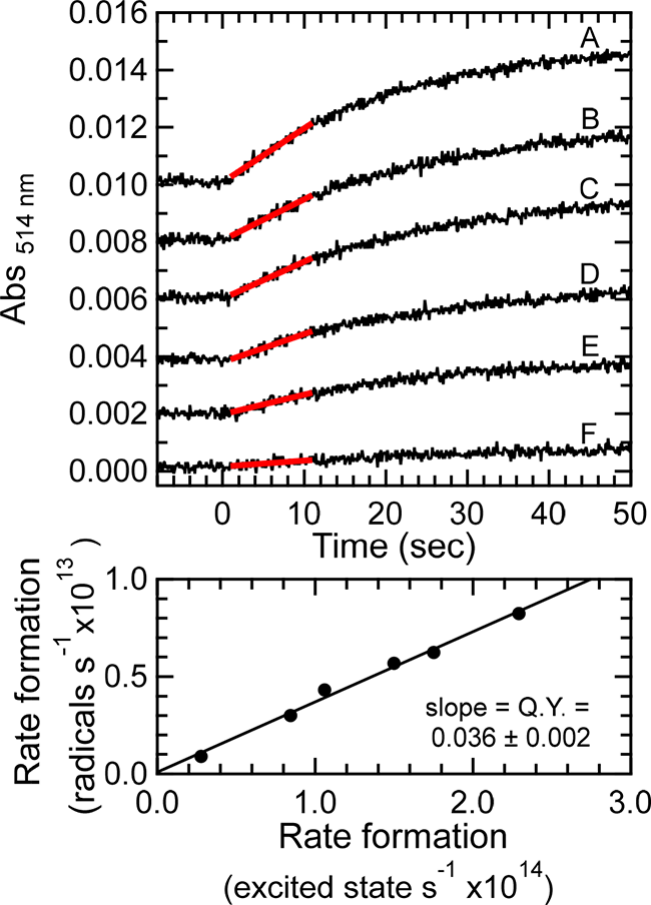
Top: Absorbance at 514 nm (same curves as Figure 5) and (red) linear least-squares fits. Inset labels A–F correspond to incident photon flux: 1.8 × 10^15^, 1.4 × 10^15^, 1.2 × 10^15^, 8.4 × 10^14^, 6.6 × 10^14^, and 2.2 × 10^14^ photons s^–1^ cm^–2^, respectively; the corresponding incident powers were 0.80, 0.63, 0.54, 0.38, 0.30, and 0.10 mW, respectively. The traces were offset (A) 0.010, (B) 0.008, (C) 0.006, (D) 0.004, and (E) 0.002 absorbance units for clarity. Bottom: Graph of rate of radical formation as a function of rate of excited-state formation (i.e., rate of absorbed light flux). The quantum yield was determined from the slope of the linear regression.

The quantum yield for formation of the neutral radical was calculated using the initial rates of radical formation at all six powers. The use of the initial rates provided an upper limit to the yield because as the concentration of neutral radical increased, the rate of formation decreased. Figure 6 shows the linear least-squares fits to the kinetic traces. The initial rise in absorbance was converted to radicals per second using the published molar absorptivity of the radical (ε_514nm_ = 2200 M^–1^ cm^–1^). In the bottom panel, a graph of the rate of radical formation as a function of the rate of excited-state formation shows a linear relationship. The quantum yield is calculated from the slope of the regression line. The quantum yield for neutral radical formation of photoexcited apoAzW48 in the presence of Co(III) is 0.036 ± 0.002, where the error arises from the linear regression. This value is much lower than the radical yield of ZnAzW48, which is greater than 0.2 under similar experimental conditions.

### Microscopic Rates from Growth and Decay Kinetics

**Figure 7:**
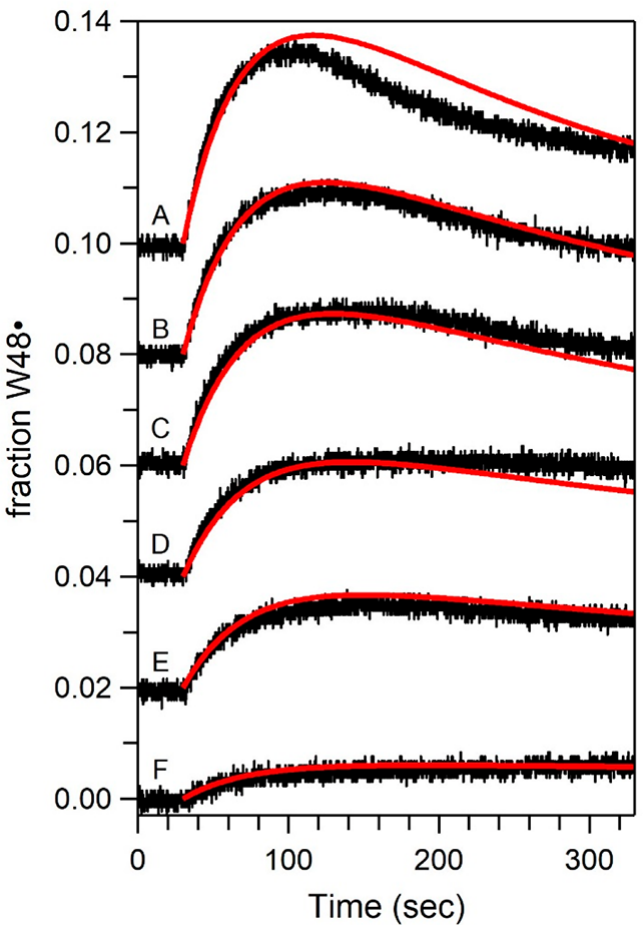
Curves from Figure 5 (curves A–F) are replotted as a fraction of W48 in apoAzW48 converted to neutral radical W48•. The curves A–F were fit simultaneously using global analysis to Scheme I (shown in red). Fixed values are ϕ_isc_ = 0.30, τ_*T*_ = 0.53 s, *k*_isc_ = 9.9 × 10^7^ s^–1^, *k*_rad_ + *k*_ic_ = 2.3 × 10^8^ s^–1^, *k*_decay_ = 0.024 s^–1^, and *k*_exc_ that corresponds to the appropriate experiment. Resulting fits yielded *k*_ET_ = 6 × 10^6^ s^–1^, *k*_deprot_ = 3 × 10^5^ s^–1^, *k*_decay_^′^ = 7 × 10^5^ s^–1^, and *k*_back_ = 2 × 10^6^ s^–1^. See text for details.

The kinetics from Figure 5 were analyzed with Scheme I in which the triplet state is the precursor to the neutral radical. Figure 7 shows the kinetics of radical formation in terms of fractional population of radical; despite the previous observation that the radical quantum yield for ZnAzW48 depends on the incident power, with decreased yields at high powers,^58^ all six powers were included in the fit. The poor fit at high power is apparent. The observation that the initial radical growth kinetics from Figure 5 is linear across the entire incident power range suggests that high incident power only affects the decay of the neutral radical. Attempts to include a power-dependence term in Scheme I were not successful (see below).

The rate constants for ET (*k*_ET_), deprotonation (*k*_deprot_), back-ET (*k*_back_), and decay of W48•^+^ (*k*_decay_^′^) were determined from global kinetic analysis of curves A–F. The following terms were fixed: *k*_excit_ was determined from experimental conditions (eq 3) with values of 0.044, 0.034, 0.029, 0.021, 0.016, and 0.0053 s^–1^ for curves A–F, respectively; the value for Φ_isc_ was 0.3 (see above); the value for *k*_deprot_ was experimentally determined as 0.024 s^–1^ (Figure 5). A global fit was performed on the six curves, and the optimized rate constants are *k*_ET_ = 6(±5) × 10^6^ s^–1^, *k*_deprot_ = 3(±1) × 10^5^ s^–1^, *k*_back_ = 2(±1) × 10^6^ s^–1^, and *k*_decay_^′^ = 7(±3) × 10^5^ s^–1^. If a lower value of Φ_isc_ = 0.2 was used, the values of *k*_deprot_ and *k*_decay_^′^ were unchanged and *k*_ET_ was similar to a value of 5 × 10^6^*s*^–1^; there was a 2.5-fold change in the value of *k*_back_ = 8 × 10^5^ s^–1^ with the lower value of Φ_isc_ = 0.2 compared to Φ_isc_ = 0.3 (Supporting Information).

### Structural Flexibility of apoAzW48 from Molecular Dynamics

**Figure 8:**
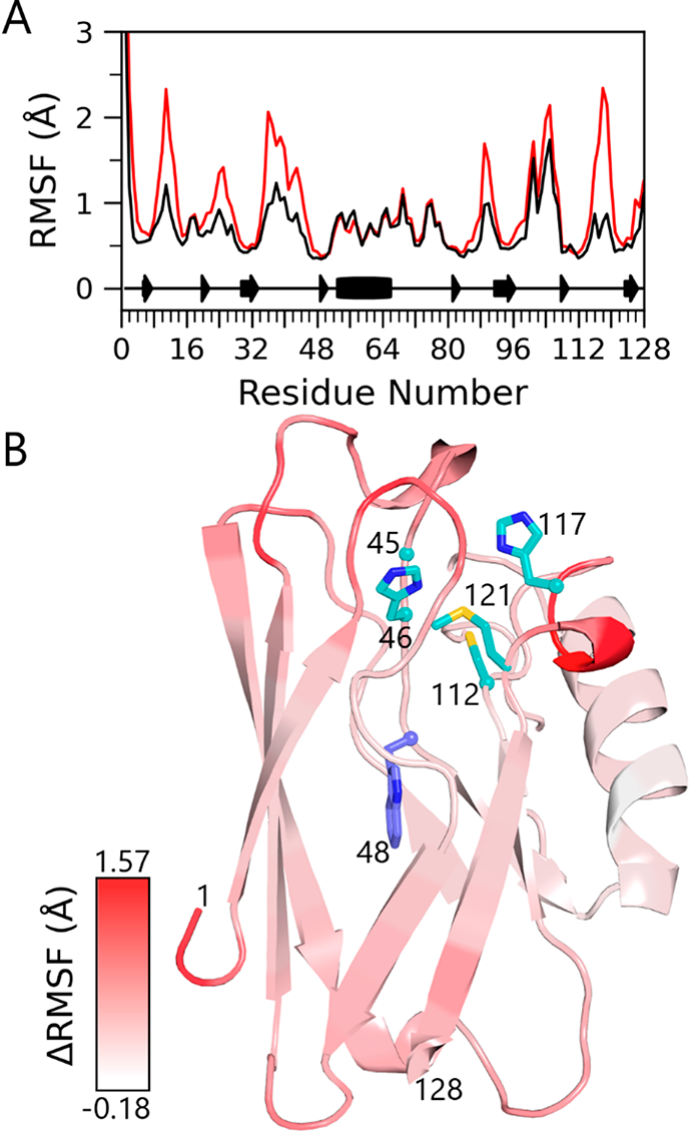
(A) Root-mean-square fluctuation (RMSF) analysis of backbone C_α_ atoms for each residue of the W48 variants of Zn-azurin (black) and apoazurin (red). Secondary structure is also indicated in the bottom portion of the graph. (B) Graph of the difference in RMSF overlaid on apoazurin. The difference in RMSF (ΔRMSF) is calculated by subtracting the ZnAzW48 RMSF from apoAzW48 RMSF. The ΔRMSF shows the effect of metal removal on the flexibility of the protein backbone. The red color illustrates the greater flexibility of apoAzW48 as predicted by the molecular dynamics simulations. Notably, with the exception of residues 56, 57, and 110, all residues in apoAzW48 had greater or equal level of fluctuations as ZnAzW48; hence, essentially all values of ΔRMSF were positive or zero. W48 is shown in purple, and the metal ligands His46, Cys112, Met121, and His117 are shown in cyan.

The structural flexibility of apoAzW48 and ZnAzW48 was investigated with molecular dynamics simulations. The apo- and Zn-variants of wild-type azurin are structurally similar;^38,43^ the average Cα root-mean-square deviation is 0.23 Å between the two structures determined from X-ray crystallography. We explored the possibility that removal of the metal changes the protein flexibility near W48, thus influencing the stability of W48•. The removal of the metal introduced larger fluctuations in apoAzW48 near the loops and metal-binding regions near residues 9–14, 35–46, and 116–120, as shown in Figure 8. The backbone torsion angles phi and psi reveal additional flexibility of residues within these regions (Supporting Information), while the backbone adjacent to W48 in both structures remained rigid as indicated by a valley of low motion in the RMSF. In the trajectories of both apo- and Zn-variants, a rare transit of a single water molecule into the hydrophobic interior was captured (Supporting Information).

## Discussion

### Triplet as the Precursor State for ET

The emission spectrum of apoAzW48 provides a clue about the precursor state for ET. In the absence of Co(III), the emission spectrum of apoAzW48 is characterized by a sharp fluorescence signal around 308 nm as well as broad phosphorescence emission around 440–520 nm. Nearly all tryptophan phosphorescence from a variety of deoxygenated proteins appears in this spectral region with the same characteristic vibronic features.^59^ When the electron acceptor Co(III) was added to a solution of apoAzW48, the emission spectrum was altered. The phosphorescence signal was eliminated, while the fluorescence intensity at 308 nm was unchanged. The elimination of phosphorescence, but not fluorescence, supports the notion that the parent triplet state is involved in the ET event in the apoAzW48···Co(III) complex.

The ability for freely diffusing molecules to decrease the tryptophan phosphorescence lifetime of several proteins has been reported.^52−54,60^ The phosphorescence quenching rate constants of azurin for oxygen have been reported as 1.3 × 10^7^ or 2.0 × 10^7^ M^–1^ s^–1^,^52,60^ and slower rate constants have been reported for the larger quenchers cinnamamide (2.5 × 10^2^ M^–1^ s^–1^)^52^ and acrylamide (1.5 M^–1^ s^–1^).^60^ These quenching rate constants for azurin are orders of magnitude smaller than for the freely diffusing molecule NATA, which has analogous quenching constants of 10^9^ M^–1^ s^–1^ for O_2_ and 10^7^ M^–1^ s^–1^ for cinnamamide.^52^ The reason for this large difference between W48 and NATA is that W48 is protected from direct encounter with a freely diffusing quencher because it is buried in a rigid protein pocket; therefore, quenching of tryptophan triplet likely involves a long-range nonradiative decay mechanism. These prior studies did not directly detect ET intermediates of tryptophan. In a different example, the ability of oxidized Fe(III)-cytochrome *c* to quench tryptophan phosphorescence lends support to an excited-state ET mechanism as a major path of nonradiative decay of the triplet.^53,54^

Excited-state ET is likely the dominant mechanism of phosphorescence quenching in the apoAzW48···Co(III) complex because other quenching mechanisms are negligible. Energy transfer via electron exchange requires contact between electron donor and acceptor;^61^ the Co(III) quencher is presumably external to the protein and more than 10 Å from W48. A dipole–dipole resonance energy transfer is also ruled out because of the weak T_1_ → S_0_ transition of the donor as well as the lack of spectral overlap between donor and acceptor. Thus, the quenched phosphorescence of apoAzW48 is mainly derived from excited-state ET from triplet state to Co(III).

The thermodynamics of ET from the triplet state is favorable because the triplet excited state is highly reducing. The one-electron reduction potential for the cation radical (Trp^•+^/Trp) is about +1.2 V versus NHE based on l-Trp;^62,63^ a recent measurement of the electrochemical potential for W48 in ZnAzW48 reported +0.952 V vs. NHE.^15^ The *E*_0,0_ energy between the ground state and the lowest-lying triplet is 3.0 eV (corresponding to 410 nm phosphorescence). These energies result in the availability of about 2 V of potential for the triplet to reduce an acceptor. Since the reduction potential of Co^3+^/Co^2+^ is 0.3 V versus NHE,^64^ there is sufficient driving force for ^3^W48* to transfer an electron to Co(III). We note that the excited-state singlet is also capable of reducing Co(III) based on the emission maximum of 308 nm (*E*_0,0_ = 4.0 eV); however, the lack of fluorescence quenching in the presence of Co(III) indicates the singlet excited state is not the dominant precursor state for ET.

In the absence of an electron acceptor, oxidation of the singlet or triplet state can still occur via direct photoionization to solvent/protein. The ionization potential of gas phase l-Trp is about 7.5 eV.^65^ With solvent stabilization of products, the ionization potential can be decreased by as much as 2.5 eV.^66^ The one-photon ionization of aqueous tryptophan and model compounds has been previously reported with 266 nm light in the solvated electron community.^67^ There is no evidence that azurin directly ejects an electron to solvent; in fact, previous experiments with solvated electron quenchers, such as N_2_O, revealed that the tryptophan radical in azurin is not formed in the presence of N_2_O.^13^ It has also been shown that disulfide bonds are effective quenchers of tryptophan triplet through electron transfer.^26^ The edge-to-edge distance between the C3–C26 disulfide bond and W48 in azurin is 13 Å, and thus could be a potential electron acceptor in the absence of the extrinsic Co(III) acceptor.

### ET Path and Comparison to Predicted ET Rate

**Figure 9:**
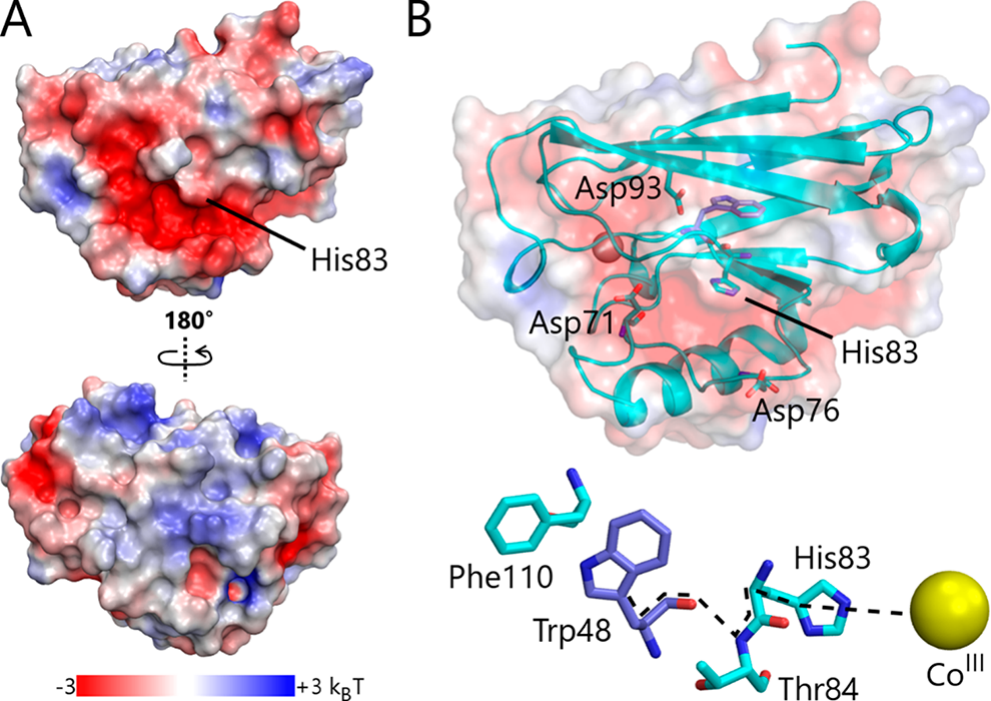
Surface representation of Zn-azurin (PDB ID: 1E67) and proposed binding site for Co(III) near His83. (A) Electrostatic potential surface calculated using PyMol/APBS. Acidic residues are shown in red, −3 *k*_B_*T*, and basic residues in blue, +3 *k*_B_*T*. The protein is rotated 180° and shown in the bottom of the panel. (B) Proposed electron transfer pathway in azurin. Acidic residues located within 10 Å of the proposed binding site for Co(III) are shown. A feasible electron transfer pathway (dotted line) from Trp48 to Co(III) via His 83 is shown in the bottom of the panel.

The ET rate depends on the intervening protein structure,^68^ and rapid ET requires a favorable electron tunneling pathway from the W48 pocket buried within the protein to the electron acceptor Co(III). As illustrated in Figure 9, positively charged Co(III) may bind onto a negative patch of protein comprised of acidic side chains near H83. A reasonable pathway to this surface histidine involves ET from the W48 backbone to T84 via a hydrogen bond, followed by transfer to H83. The Co(III) acceptor is expected to be coupled into this pathway through an ammine ligand to the histidine imidazole by a through-space jump. The observed ET rate will reflect the dominant route, or an average of rates should more than one pathway be possible.

The measured ET rate constant in the Co(III)-complex was determined by fitting the data of the neutral radical, and compared to a predicted rate. The maximum electron tunneling rate was calculated using the relation *k*_max_ = 10^13^ s^–1^ exp{−β(*R* – 3 Å)} for donor and acceptor separated by protein medium, where β is the distance-decay constant and *R* is the donor–acceptor separation.^69^ In the proposed Co(III)-complex, the π edge-metal separation is estimated to be ∼15.6 Å; this includes a 10.6 Å spatial separation between C_γ_ W48 and N_ε2_ H83, an estimated 3.0 Å through-space distance to acceptor, and a 2.0 Å bond between amine N and Co from the cobalt pentaaminechloride crystal structure.^70^ Using an average value of β for Ru-azurins of 1.1 Å^–1^, based on experiment,^69^ yields a maximum ET rate of 1 × 10^7^ s^–1^. This estimate of the ET rate assumes preassociation of Co(III) with the protein and requires the acceptor to remain localized on the surface during the ET event. An increase in the donor–acceptor separation at the interface is expected to decrease the rate. However, interfacial water molecules can mediate ET by bridging a connection to the pathway with hydrogen bonds, thereby providing a means for the observed ET rate to remain close to the maximum rate.^71−73^

The predicted ET rate constant of 1 × 10^7^ s^–1^ is comparable to the experimentally determined rate constant of 6 × 10^6^ s^–1^. This rate constant is much faster than the decay rate constant of the triplet state of 1.9 s^–1^, indicating that in the presence of an electron acceptor such as Co(III), the triplet state would be strongly quenched, consistent with the results presented here (Figure 1). However, the ET rate is slower than the decay rate constant of the fluorescent state of 3.3 × 10^8^ s^–1^, supporting the notion that oxidation occurs primarily from the triplet state. The predicted rate constant is also faster than the calculated deprotonation rate constant of 3 × 10^5^ s^–1^. Hence, the rate-limiting step to formation of the neutral radical appears to be deprotonation. These kinetic results agree with a stepwise proton-coupled ET mechanism in which the cation radical is first generated from the triplet state followed by proton transfer. In the global analysis of the kinetics of the neutral radical, the notably poor fit to curve A (high-power excitation) as well as modest fit to the other high-power data sets nonetheless yielded reasonable values for ET, deprotonation, and decay of radical cation. Attempts were made to compensate for poor fits by including parameters for power dependence in the cation and neutral radical decay; however, these attempts resulted in worse fits overall with increased fitting error and did not yield new insights.

### Instability of the Neutral Radical in apoAzW48

This study examined tryptophan neutral radical generated in apoAzW48···Co(III) complexes. In the tyrosine deficient mutant of azurin, the native hole transfer pathway is disrupted, allowing the formation and decay kinetics of the radical to be characterized. As shown in Figure 5, the neutral radical decay lifetime of W48• in apoAzW48 is 41 s. Compared to its apo-counterpart, the stability of W48• in ZnAzW48 is quite remarkable with a decay lifetime of 7.3 h.^13^ The quantum yield of neutral radical formation for apoAzW48 is also significantly decreased by more than 6-fold to 0.036 relative to ZnAzW48. This substantial difference in decay kinetics and quantum yield is consistent with an alteration of the dynamics of the protein and not modification of the local environment of W48. The similarity of the W48 protein pocket for Zn- and apoazurin are evident in the fluorescence spectra. The blue-shifted fluorescence of W48 reports on the local hydrophobic environment, and in both apo- and ZnAzW48, the fluorescence is unchanged with a maximum at 308 nm. The phosphorescence quantum yields of apoAzW48 (0.015) and ZnAzW48 (0.018 based on experiments in our lab) are also very similar. Indeed, the X-ray crystal structures^38,43^ of both forms show that the three-dimensional arrangement of heavy atoms surrounding tryptophan is almost identical; the root-mean-square difference of C_α_ atoms in W48 is less than 0.23 Å between these two structures.

Protein flexibility appears to be key for understanding the instability of the radical. In apoazurin, a water molecule can occupy the metal binding site, preventing rearrangement of the side chains that usually form the metal ligands.^38^ However, it was reported that this water is labile, and when absent from the metal-binding site, the side chains rearrange. The molecular dynamics simulations support rearrangement of the metal-binding ligands, showing the opening of a cavity between residues His46 and His117 that allows the entry of water molecules (Supporting Information). This dynamic exchange of water in the metal-binding cavity and subsequent rearrangement of the ligating (redox-active) residues may be responsible for the decreased stability of W48• in apoAzW48. Native azurin is well-known for its unusually high resistance to thermal and chemical denaturation, and incorporation of a metal ion considerably increases the thermodynamic stability of apoazurin by as much as 23–24 kJ/mol.^16−18^ These results suggest that a metal-induced stabilization may create a rigid pocket that reduces the flexibility of protein side chains and backbone, thereby impacting the stability of W48•. In the absence of a metal center, the protein is more dynamic with rearranged ligating residues and thus, the neutral radical is destabilized and exhibits a significantly reduced lifetime of seconds as opposed to hours.

## Conclusion

It is known that the rigid hydrophobic pocket surrounding W48 enables a long-lived triplet state in azurin. Results from this study suggest that this triplet state is the parent state responsible for ET from tryptophan to an exogenous electron acceptor in apoAzW48. Kinetic modeling revealed reasonable rate constants for ET (6 × 10^6^ s^–1^) and subsequent deprotonation (3 × 10^5^ s^–1^). However, the radical quantum yield and radical lifetime for W48• in apoAzW48 are low compared to the metalated species, ZnAzW48. This difference suggests that the presence of a metal center significantly impacts the generation and stability of the radical. MD simulations point to the enhanced dynamics and flexibility of the metal binding pocket in apoAzW48 as a contributing factor for the suboptimal generation and stability of W48•.

## Supporting Information Available

Equations for the kinetic model; schematic of spectrophotometer and photolysis beam paths; emission spectra of apoAzW48 and NATA; absorption spectra of apoAzW48 in the absence and presence of 405 nm light; kinetic fits using Φ_isc_ = 0.2; Ramachandran plots; molecular dynamics simulation results (PDF)
