# Supplementary material for: Role of the Triplet State and Protein Dynamics in the Formation and Stability of the Tryptophan Radical in an Apoazurin Mutant
Source: J Phys Chem B. 2022 Aug 17;126(36):6751–61. doi: 10.1021/acs.jpcb.2c02441 (PMC9483921; doi:10.1021/acs.jpcb.2c02441)
Supplement: Supplementary file 1 — jp2c02441_si_001.pdf [file jp2c02441_si_001.pdf]

Supporting Information

Role of the triplet state and protein dynamics in the formation and stability of the tryptophan radical in an apoazurin mutant

Ignacio López-Peña, Christopher T. Lee[1], Joel J. Rivera, and Judy E. Kim[*]

Department of Chemistry and Biochemistry, University of California at San Diego, La Jolla, CA

[1]Currently at the Department of Mechanical and Aerospace Engineering, University of California at San Diego, La Jolla, CA

**Corresponding author email: judyk@ucsd.edu*

The differential equations for Scheme I are:

$$\frac{d[\mathrm{W48}]}{dt} = -k_{excit}[\mathrm{W48}] + (k_{rad} + k_{ic})[W48^{*}] + \left(\frac{1}{\tau_T}\right)[^{3}\mathrm{W48}^{*}] + k_{back}[\mathrm{W48}\bullet^{+}]$$

$$\frac{d[\mathrm{W48}^{*}]}{dt} = k_{excit}[W48] - (k_{rad} + k_{ic})[W48^{*}] - k_{isc}[^{3}\mathrm{W48}^{*}]$$

$$\frac{d[^{3}\mathrm{W48}^{*}]}{dt} = k_{isc}[\mathrm{W48}^{*}] - \left(\frac{1}{\tau_T}\right)[^{3}\mathrm{W48}^{*}] - k_{ET}[^{3}\mathrm{W48}^{*}]$$

$$\frac{d[\mathrm{W48}\bullet^{+}]}{dt} = k_{ET}[^{3}\mathrm{W48}^{*}] - k_{deprot}[\mathrm{W48}\bullet^{+}] - k'_{decay}[\mathrm{W48}\bullet^{+}] - k_{back}[\mathrm{W48}\bullet^{+}]$$

$$\frac{d[\mathrm{W48}\bullet]}{dt} = k_{deprot}[\mathrm{W48}\bullet^{+}] - k_{decay}[\mathrm{W48}\bullet]$$

$$\frac{d[\mathrm{W48}^{\mathrm{X}}]}{dt} = k'_{decay}[\mathrm{W48}\bullet^{+}] + k_{decay}[\mathrm{W48}\bullet]$$

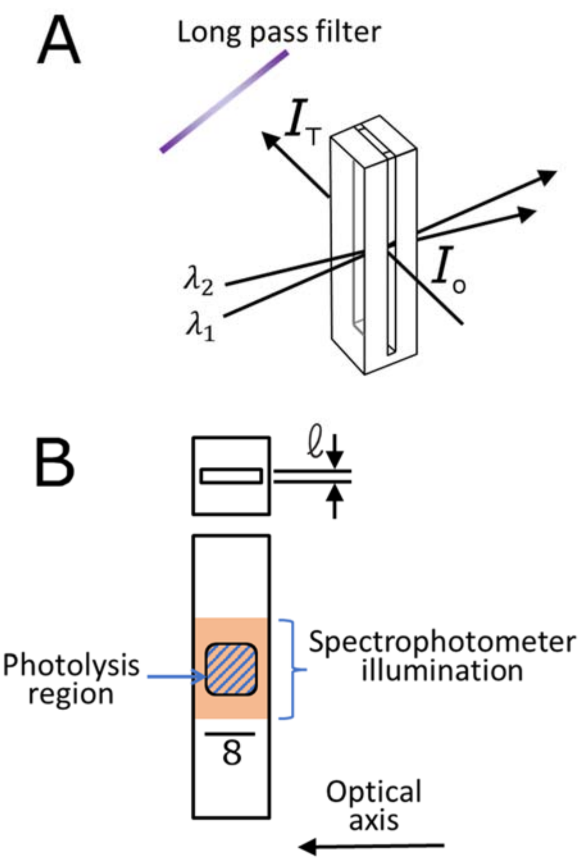

Figure S1: Schematic of spectrophotometer and photolysis beam paths. Panel A: Actinic light where wavelengths λ_1 (280 nm) and/or λ_2 (405 nm) entered the sample orthogonal to the optical axis of the UV-Vis spectrophotometer. Panel B: The beams (λ_1 and/or λ_2) overlapped inside a volume defined by the 8 mm illuminated spot on the cuvette and pathlength of ℓ=2 mm. The incident beam of the spectrophotometer (I_0) had a typical width of about 1.8 mm, and height of 16 mm.

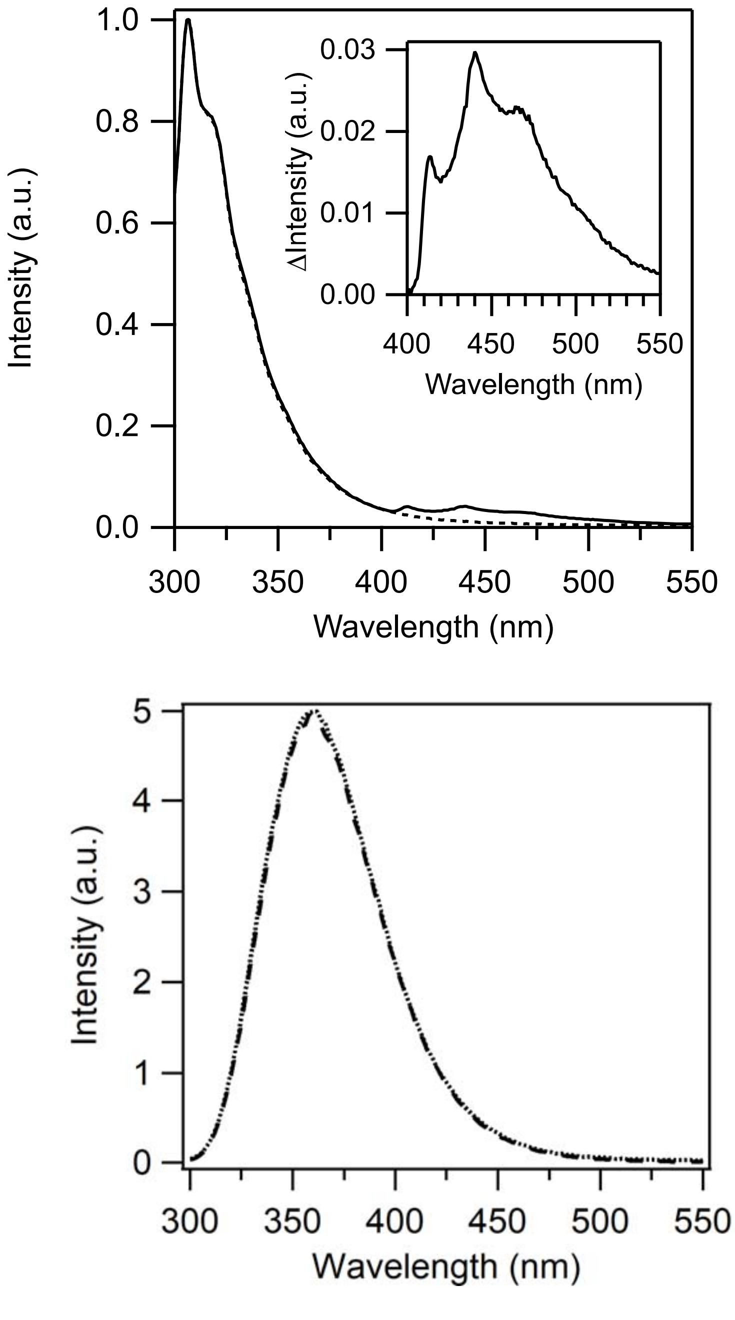

Figure S2: Top: Room-temperature emission spectra of 50 μM apoAzW48 in deoxygenated 20 mM phosphate buffer (pH 7.3) in the absence (solid line) and presence (dashed line) of 100 μM $[Co(NH_3)_5Cl]^{2+}$. Excitation was at 292 nm. The spectra were normalized for peak fluorescence intensity. The inset shows the difference spectrum of the emission in the absence of $[Co(NH_3)_5Cl]^{2+}$ minus the emission in the presence of $[Co(NH_3)_5Cl]^{2+}$. The remaining difference feature is tryptophan phosphorescence. Bottom: Fluorescence spectra of NATA in air in the absence (dashed) and presence (dotted) of $[Co(NH_3)_5Cl]^{2+}$. The NATA spectra have not been normalized (only signal from buffer has been subtracted).

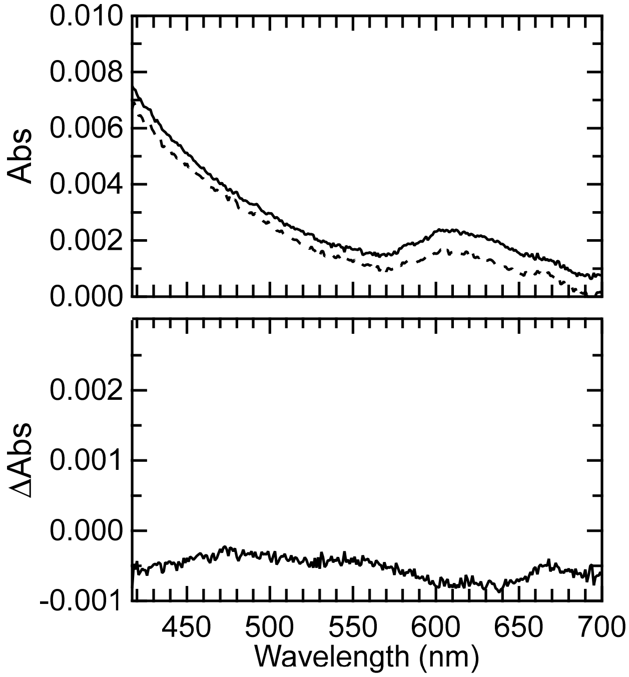

Figure S3: Top: Absorption spectrum of 50 μM apoAzW48 in deoxygenated 20 mM phosphate buffer (pH 7.3) before (dashed line) and during 405 nm irradiation (solid line). The electron acceptor $[Co(NH_3)_5Cl]^{2+}$ was absent from the sample. Bottom: the difference spectrum after subtraction of the pre-irradiation spectrum from the continuous-irradiation spectrum. The difference spectrum shows a baseline offset caused by 405 nm illumination of the sample.

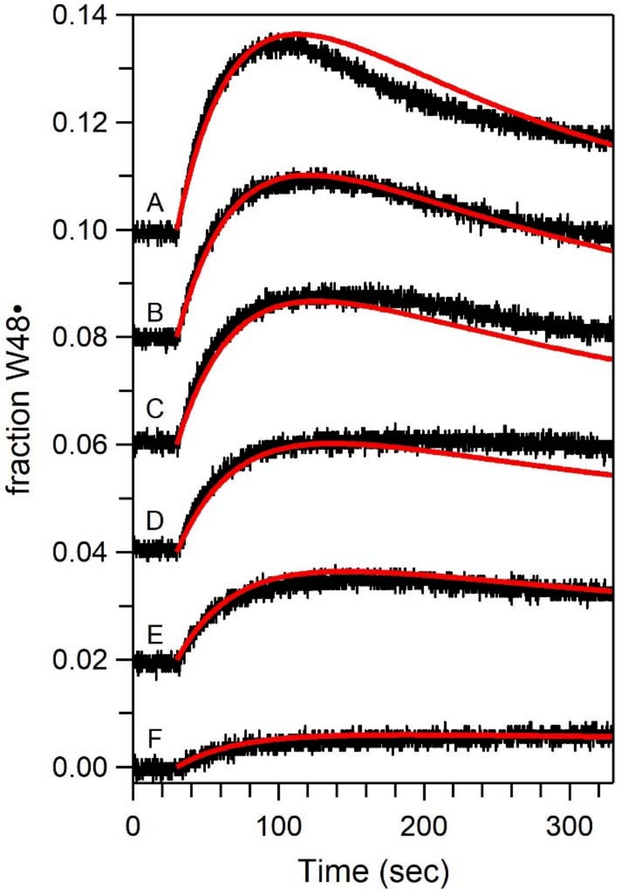

Figure S4: The curves from Figure 5 (curves A, B, C, D, E, F) were replotted as fraction of W48 in apoAzW48 converted to neutral radical W48•. The curves A, B, C, D, E, F were fit simultaneously using global analysis to Scheme I (shown in red). Fixed values are $\phi_{isc} = 0.20$, $\tau_T = 0.53\ s$, $k_{isc} = 9.9 \times 10^7\ s^{-1}$, $k_{rad} + k_{ic} = 2.3 \times 10^8\ s^{-1}$, $k_{decay} = 0.024\ s^{-1}$, and k_{exc} that corresponds to the appropriate experiment. Resulting fits yielded $k_{ET} = 5 \times 10^6\ s^{-1}$, $k_{deprot} = 3 \times 10^5\ s^{-1}$, $k'_{decay} = 7 \times 10^5\ s^{-1}$, and $k_{back} = 8 \times 10^5\ s^{-1}$, See text for details.

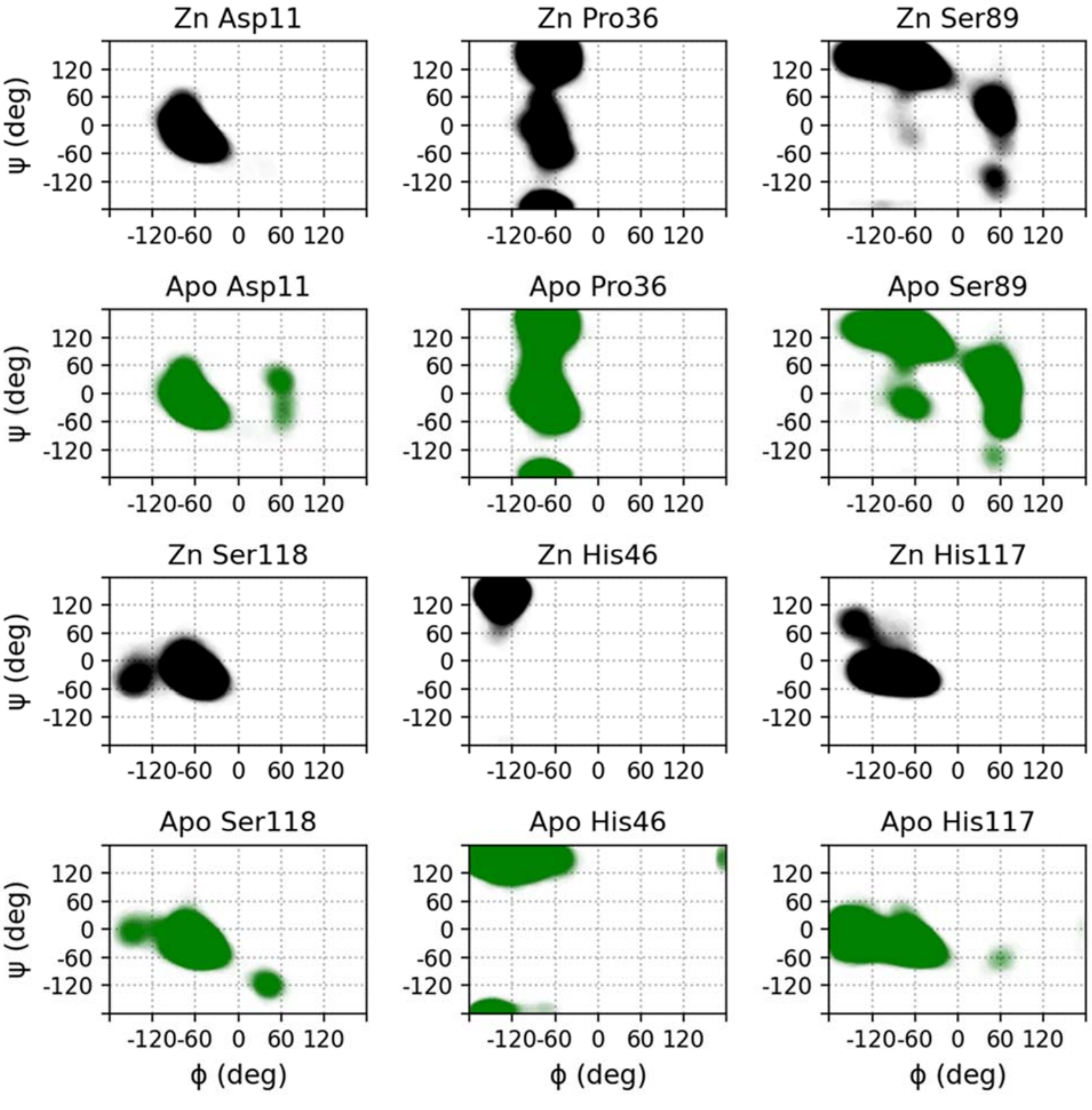

Figure S5: Ramachandran plot of accessible backbone phi/psi dihedral angles for residues located in regions with large fluctuations as calculated from molecular dynamics trajectories using PYTRAJ. ZnAzW48 (black), and apoAzW48 (green).

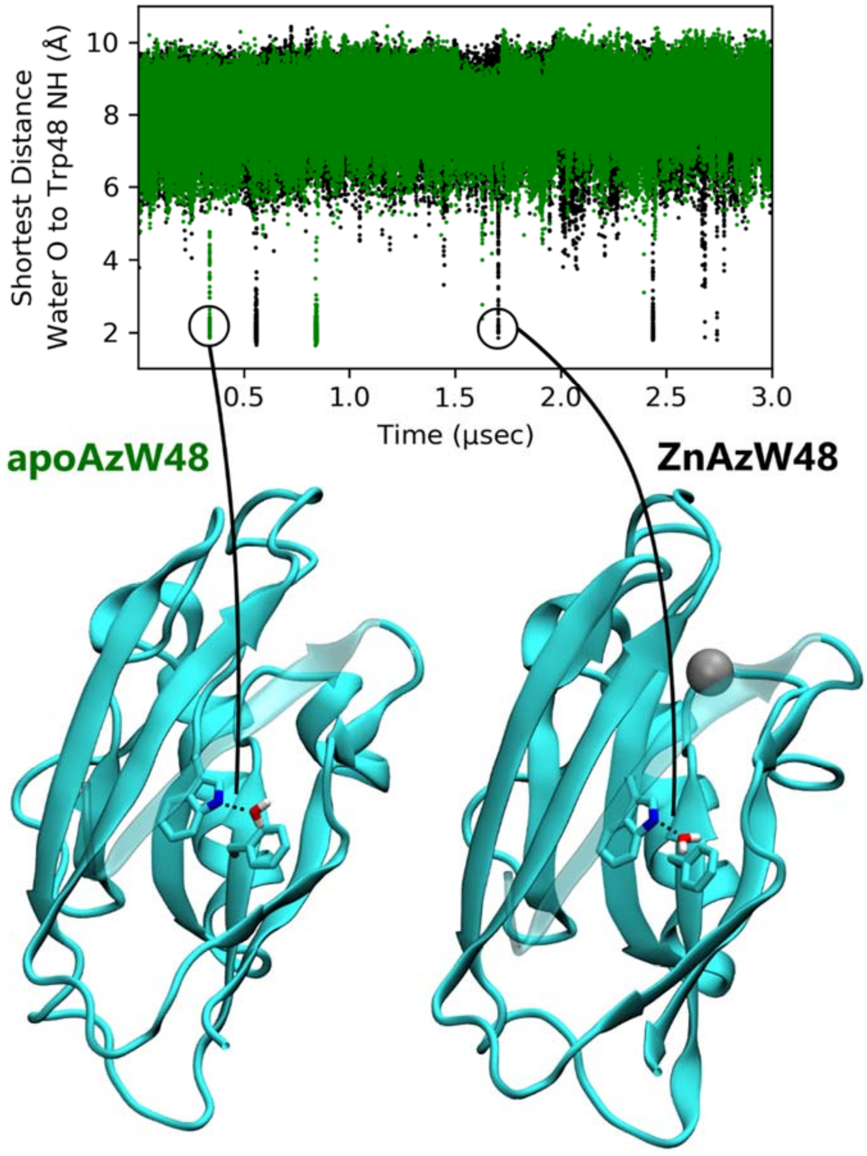

Figure S6. Results from the 3 μsec molecular dynamics simulations that show the shortest distance between the nearest water oxygen and W48 NH of apoAzW48 (green) and ZnAzW48 (black). There are few transits of a water molecule into the hydrophobic interior of the protein surrounding W48 and F110.

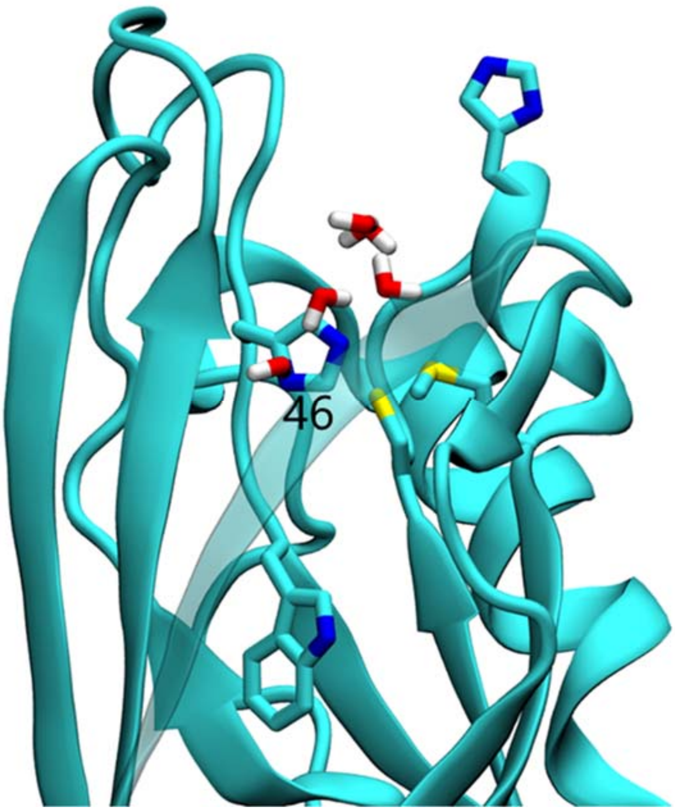

Figure S7. Water molecules within 5 Å of His46 inside the metal-binding cavity of apoAzW48 taken from a single-frame of the 3 μsec molecular dynamics simulation of apoAzW48. Trp48, Cys112, Met121, and His117 are also shown as sticks.
